# Ag Nanoparticles (Ag NM300K) in the Terrestrial Environment: Effects at Population and Cellular Level in *Folsomia candida* (Collembola)

**DOI:** 10.3390/ijerph121012530

**Published:** 2015-10-09

**Authors:** Luís André Mendes, Vera L. Maria, Janeck J. Scott-Fordsmand, Mónica J. B. Amorim

**Affiliations:** 1 Department of Biology & CESAM, University of Aveiro, Aveiro 3810-193, Portugal; E-Mails: lamendes@ua.pt (L.A.M.); vmaria@ua.pt (V.L.M.); 2 Department of Bioscience, Aarhus University, Vejlsovej 25, PO BOX 314, Silkeborg DK-8600, Denmark; E-Mail: jsf@bios.au.dk

**Keywords:** antioxidant defenses, mechanisms of response, soil invertebrates

## Abstract

The effects of nanomaterials have been primarily assessed based on standard ecotoxicity guidelines. However, by adapting alternative measures the information gained could be enhanced considerably, e.g., studies should focus on more mechanistic approaches. Here, the environmental risk posed by the presence of silver nanoparticles (Ag NM300K) in soil was investigated, anchoring population and cellular level effects, *i.e.*, survival, reproduction (28 days) and oxidative stress markers (0, 2, 4, 6, 10 days). The standard species *Folsomia candida* was used. Measured markers included catalase (CAT), glutathione reductase (GR), glutathione S-transferase (GST), total glutathione (TG), metallothionein (MT) and lipid peroxidation (LPO). Results showed that AgNO_3_ was more toxic than AgNPs at the population level: reproduction EC_20_ and EC_50_ was ca. 2 and 4 times lower, respectively. At the cellular level Correspondence Analysis showed a clear separation between AgNO_3_ and AgNP throughout time. Results showed differences in the mechanisms, indicating a combined effect of released Ag^+^ (MT and GST) and of AgNPs (CAT, GR, TG, LPO). Hence, clear advantages from mechanistic approaches are shown, but also that time is of importance when measuring such responses.

## 1. Introduction

The effects of nanomaterials have been primarily assessed via the use of standard ecotoxicity guidelines, although, there are evidences that adaptations and alternatives should be considered, e.g., the required exposure time should be adjusted [1]. The use of more mechanistic based studies can provide many advantages supporting the present standard tests, e.g., understanding the mode of action can be used as a background for extrapolating from short to long-term effects, an issue which has high priority [2].

Silver nanoparticles (AgNPs), which are widely used due to their bactericidal properties, have also been reported toxic for a diverse range of organisms, e.g., for soil invertebrates such as *Eisenia fetida* [1,3,4], *Enchytraeus albidus* [5], *Eisenia andrei* [6], *Porcellio scaber* [7] and *Folsomia candida* [8]. One of the known mechanisms related to Ag toxicity is the induction of oxidative stress. This process is the result of the increase of reactive oxygen species (ROS) in the organism, causing an unbalance and activation of the antioxidant defense mechanisms [9,10]. These include the activation of several enzymatic and non-enzymatic proteins, such as catalase (CAT), glutathione reductase (GR) or metallothioneins (MTs). The methodology to measure such markers has been optimized for various soil organisms, including *Folsomia candida* [11], *Enchytraeus albidus* [12] or *Eisenia fetida* [1].

Here, the environmental effect of silver nanoparticles (Ag NM300K) in soil was investigated, anchoring population and cellular level effects, *i.e.*, survival, reproduction (standard test, 28 days) after which the oxidative stress markers were evaluated at the reproduction Effect Concentration that Causes 50% Reduction (EC_50_), along an exposure time series: 0, 2, 4, 6, 10 days. The species *Folsomia candida* (Collembola) was used as test species. Collembolans have been widely used to assess the environmental impact of e.g., organic chemicals [13], pesticides [14], metals [15], mixtures [16] or nanomaterials [8,17,18]. The markers used were catalase (CAT), glutathione reductase (GR), glutathione S-transferase (GST), total glutathione (TG), metallothionein (MT) and lipid peroxidation (LPO).

## 2. Experimental Section

### 2.1. Test Organism

*Folsomia candida* (Collembola) was used as test species [19]. Cultures were maintained in laboratory on a moist substrate of Paris plaster and activated charcoal (8:1 ratio) at 19 ± 1 °C, under a photoperiod regime of 16:8 (light:dark). The organisms were fed once a week with dried baker’s yeast (*Saccharomyces cerevisae*). Organisms of synchronized age (10–12 days) were used for the experiments, as within the standard protocol. 

### 2.2. Test Materials

Test materials included Ag salt and Ag nanomaterial. The AgNO_3_ (high-grade, 98.5%–99.9% purity) was purchased from Sigma-Aldrich (St. Louis, MO, USA). The silver nanoparticles (AgNPs) used were the standard reference materials Ag NM300K from the European Commission Joint Research Centre (JRC), fully characterized [20]. The Ag NM300K is dispersed in 4% polyoxyethylene glycerol triolaete and polyoxyethylene (20) sorbitan monolaurate (Tween 20), thus the dispersant was also tested alone.

### 2.3. Test Soil and Spiking

Natural standard LUFA (Landesanstalt für Umwelt und Forschung) soil 2.2 (Speyer, Germany) was used. The general soil properties are as follow: pH = 5.5, organic carbon = 1.77%, cation exchange capacity = 10.1 meg/100 g, and grain size distribution of 7.3% clay; 13.8% silt and 78.9% sand.

Ag was spiked as aqueous solution and serially diluted. The soil was pre-moistened before spiking, to obtain a final water holding capacity of 50%, and aged for 72 h before test start. For Ag NM300K, spiking was done individually for each replicate. For AgNO_3_, the various replicates per treatment were spiked together and then divided into each test vessel as within standard. Concentration range for AgNO_3_ was: 0, 64, 100, 130, 320, 640 mg Ag/kg soil dry weight (DW) and for AgNP was: 0, 64, 130, 220, 320, 640 mg Ag/kg soil DW. A control dispersant was used adding the same volume as used with the highest concentration of Ag NM300K to assess the effect of the dispersant alone. Test concentration used for the biomarker exposure corresponded to the reproduction EC_50_ (value selected within the confidence interval). The choice of this EC50 was based on its relevance in Risk Assessment and linkage to reproduction chronic effects. Moreover, the tested concentration should be sub-lethal to ensure organisms’ survival for sampling and for mechanistic studies before narcosis (not relevant for biomarkers). 

### 2.4. Test Procedure 

#### 2.4.1. Population Level—Standard Reproduction Test 

Tests followed the standard reproduction ISO (International Standardization Organization) test guideline for collembolans [19]. In short, 10 juveniles (10–12 days) were transferred to the test vessels containing the soil. Four replicates were used per treatment. Test ran at 20 °C and 16:8 h (light:dark) photoperiod; food supply and water was replenished every week. Reproduction and adult survival were assessed after 28 days by flotation method to count the number of adults and juveniles.

#### 2.4.2. Cellular Level—Oxidative Stress Biomarkers

Procedures followed the same as in the standard guideline [19] with adaptations [11]. A pool of 50 juveniles of 13–14 days was used as a replicate. Ten (10) replicates (five for MT measurements plus five for the other markers) per treatment were performed. Exposure period included samplings at 0, 2, 4, 6, 10 days. At each sampling time organisms were extracted by flotation, transferred to plaster to absorb the excess water and pooled into microtubes, weighted and snap-frozen in liquid nitrogen, being stored at −80°C until further analysis. Five replicates per condition were used for metallothionein (MT) quantification and the other five for the rest of all biochemical analysis, *i.e.*, catalase (CAT), glutathione reductase (GR), glutathione S-transferase (GST), total glutathione (TG) and lipid peroxidation (LPO). Biomarkers measurements were performed following the procedures as described in Maria *et al.* [11]. 

### 2.5. Data analysis

One-way ANOVA and Post Hoc Dunnett’s test was used to identify significant differences between control and treatments [21]. The effect concentrations (ECx) were calculated using the Toxicity Relationship Analysis Program (TRAP 1.21) applying the 2-parameters Logistic model. To assess differences between control and control dispersant a t-test (*p* < 0.05) was used. 

Multivariate analysis was done using Correspondence Analysis (CA) including all treatments. The analysis was performed using the software SAS Enterprise Guide 5.1 [22]. To compensate for the different scales of the biomarkers, the response was normalised before use, several different normalisation methods were tested overall giving the same pattern; the present normalisation was based on averaging in relation to the mean.

## 3. Results

### 3.1. Materials Characterization

The silver nanoparticles (AgNPs) used were the standard reference materials Ag NM300K from the European Commission Joint Research Centre (JRC), fully characterized [20]. In short, Ag NM300K are spherical and consist of a colloidal dispersion with a nominal silver content of 10.2 w/w %, dispersed in 4% w/w of polyoxyethylene glycerol trioleate and polyoxyethylene (20) sorbitan monolaurate (Tween 20), having > 99% number of particles with a nominal size of about 15 nm, with no coating. Transmisson Electron Microscopy (TEM) indicated a size of 17 ± 8 nm. Smaller nanoparticles of about 5 nm are also present.

### 3.2. Biological Characterization

#### Population Level—Standard Reproduction Test

**Figure 1:**
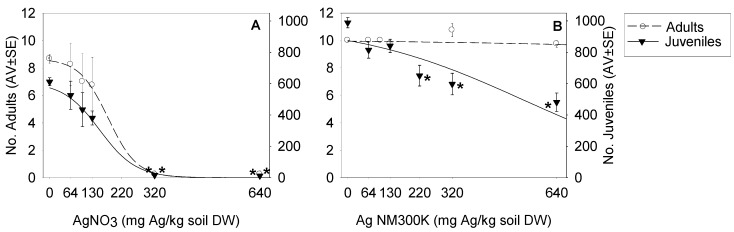
Survival (number of adults) and reproduction (number of juveniles) for *Folsomia candida* when exposed in LUFA 2.2 soil to (**A**) AgNO_3_ and (**B**) Ag NM300K. Results are expressed as average ± standard error (Av ± SE) (*n* = 4). *: Dunnett’s (*p* < 0.05) for differences between control and treatments. Lines represent the model fit to data.

The tests validity criteria were fulfilled, *i.e.*, less than 20% mortality, >100 juveniles per replicate and the coefficient of variation < 30%. Results can be observed in Figure 1. For Ag NM300K no differences between control and control dispersant were observed (*p* > 0.05): Adult survival (average (AV) ± standard error (SE)): 10 ± 0; Juvenile Reproduction (AV±SE): 977 ± 50 and 1004 ± 47, respectively, hence data was modeled pooling both controls. A dose response effect was observed, with Ag NM300K being less toxic than AgNO_3_. The estimated ECx values can be seen in Table 1.

**Table 1:** Effect Concentrations (ECx) for survival and reproduction of *Folsomia candida* when exposed to AgNO_3_ and AgNPs (Ag NM300K). n.d.: not determined. n.e.: no effect (95% Confidence Intervals). EC_10_, _20_, _50_, _80_: Concentration that causes 10%, 20%, 50%, 80% Effect, respectively. S: relative slope estimated at EC_50_, Y0: Average control value (average of control values for survival and reproduction).

Test Materials	EC_10_ (mg/kg)	EC_20_ (mg/kg)	EC_50_ (mg/kg)	EC_80_ (mg/kg)	Model and Parameters
Survival					
AgNO_3_	82 (20–162)	118 (62–174)	179 (77–280)	240 (57–422)	Logistic 2 parameters (S:0.0057; Y0:8.7)
Ag NM300K	n.e.	n.e.	n.e.	n.e.	–
Reproduction					
AgNO_3_	31 (−35–97)	76 (36–115)	152 (108–196)	228 (134–324)	Logistic 2 parameters (S:0.0045; Y0:610.0)
Ag NM300K	n.d.	173 (70–277)	540 (412–667)	906 (653–1159)	Logistic 2 parameters (S:0.0009; Y0:988.3)

### 3.3. Cellular Level—Oxidative Stress Biomarkers

#### 3.3.1. Univariate Analysis

**Figure 2:**
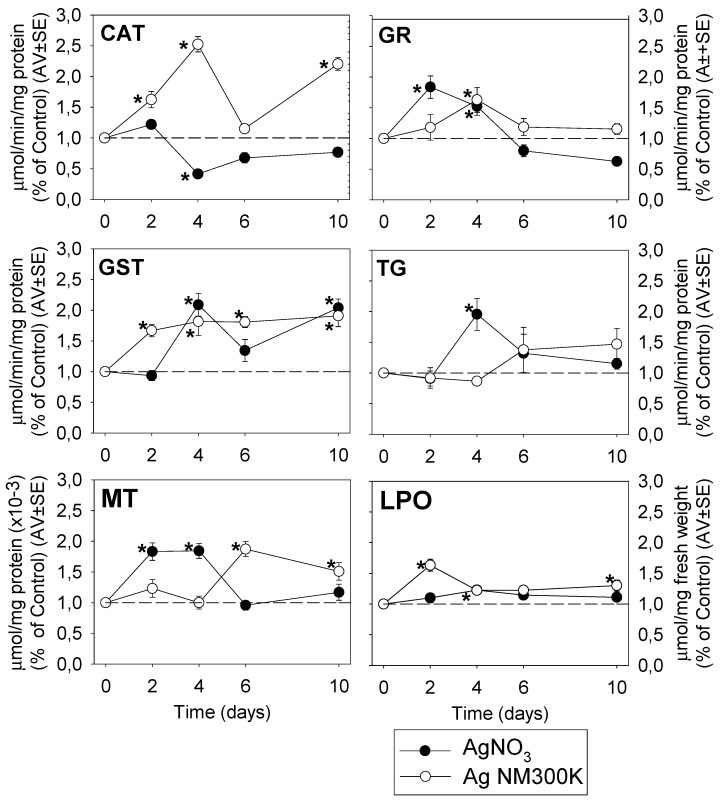
Oxidative stress biomarker results for *Folsomia candida* when exposed in LUFA 2.2 soil to the reproduction EC_50_ of AgNO_3_ (black dots) and Ag NM 300K (white dots). Results are expressed as % and normalized to the respective controls (water and dispersant) mean values ± standard error (Av ± SE) (*n* = 5). Dotted horizontal line represents the control reference, *i.e.*, 100%. CAT: Catalase; GR: Glutathione Reductase (GR), GST: Glutathione S-Transferase, TG: Total Glutathione; MT: Metallothionein; LPO: Lipid Peroxidation; *: Dunnett’s test (*p* < 0.05) for differences between control and treatments.

For Ag NM300K the control dispersant was used as a reference because for LPO, TG and GR measurements there were differences (*p* < 0.05) between control and control dispersant (Figure 2).

For AgNO_3_, CAT activity decreased after 4 days exposure (*p* < 0.05) (0.4-fold to control), maintaining a tendency of low values in the remaining exposure time. GR activity shows an increase after 2 and 4 days (*p* < 0.05) followed by a decrease to levels lower than control at 6 and 10 days exposure. MT shows a similar pattern. GST activity shows an increase-decrease-increase behaviour at 4 (*p* < 0.05), 6 and 10 (*p* < 0.05) days respectively and TG increased only at day 4 (*p* < 0.05). Significant increase in LPO levels was observed at day 4 (1.2-fold, *p* < 0.05).

For Ag NM300K CAT activity was higher after 2, 4 and 10 days (*p* < 0.05), having a decrease tendency at day 6 (to levels similar to control). GST activity showed an increase (*p* < 0.05) up to day 4 and then continued on same levels until day 10 (*p* < 0.05). GR increased only after 4 days (*p* < 0.05). TG levels were lower than control after 4 days and superior at 10 days. MT levels increased after 6 days (*p* < 0.05), maintaining the higher level at day 10. LPO increased at day 2 (*p* < 0.05), after which it decreased to be increased again at day 10 (*p* < 0.05). 

#### 3.3.2. Multivariate Analysis

**Figure 3:**
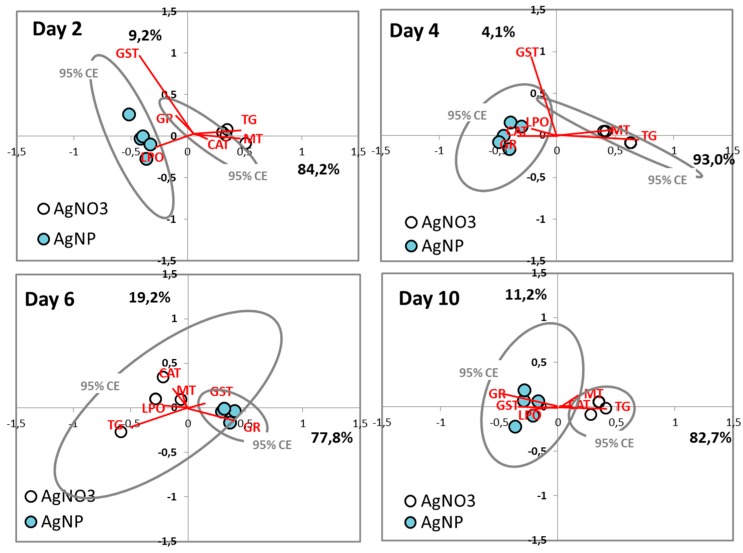
Correspondence Analysis (CA) of data from *Folsomia candida* exposed to AgNP (Ag NM300K) [640 mg Ag/kg soil] and AgNO_3_ [145 mg Ag/kg soil], as sampled at 0-2-4-6-10 days, in terms of Catalase (CAT), Glutathione Peroxidase (GPx), Glutathione S-Transferase (GST), Glutathione Reductase (GR), Total Glutathione (TG), Metallothionein (MT) and Lipid Peroxidation (LPO). Percentage (%) explanatory power is added for each axis. All time points showed significant differences (discriminant analysis), the day 6 time point shows largest overlap of the two confidence ellipse, which show the difference here is the least.

The multivariate analysis of the data (Correspondence Analysis) enabled an identification of the overall differences between the AgNO_3_ and AgNP exposures (Figure 3), with a mainly clear separation between the AgNO_3_ and AgNP throughout time. [It should be noted that whereas Figure 2 shows mean values and standard errors, the multivariate plot displays the individual replicates]. It is seen that LPO and GST were primarily associated with AgNP and MT and TG associated with AgNO_3_, hence these markers would be the primary identifiers of different exposures. In the later exposure stages (10 days) the GR was most pronounced for the AgNP exposure, when compared to AgNO_3_ exposure. The larger confidence ellipse (compared to others) related to the AgNO_3_ at day 6, seem to be related to one replicate having a relative high (again compared to the others) TG. 

## 4. Discussion

### 4.1. Population Level

Results showed that AgNO_3_ displayed higher toxicity than Ag NM300K for *Folsomia candida*, with increasing difference with higher concentration (EC_20_ to EC_80_). For AgNO_3_, the Effect Concentration (EC) values were within the obtained confidence interval as found by Waalewijn-Kool *et al.* [8] for *F. candida* tested under the same conditions. The same authors tested other AgNP (paraffin coated, 3–8 nm, water dispersed) and found no effect on survival or reproduction up to 673 mg Ag/kg soil DW. As concluded by the authors, the internal Ag concentrations for *F. candida* could not explain the higher toxicity of AgNO_3_ compared to AgNPs; it has been suggested that the higher internal Ag in *F. candida* exposed to AgNPs could be because these are taken up on the particulate form. Unlike ZnO NPs [18,23], porewater concentrations could not explain the toxicity of AgNPs. It seems that AgNPs aggregation and sorption to soil parts reduces dissolution. The fate of AgNPs in soil has been reported complex, with e.g., soil type, dissolution (rate), oxidation, nanoparticle size and the type of coating influencing the availability of Ag [8]. For other invertebrates, oligochaete studies has shown that AgNO_3_ was more toxic than AgNPs [5,6,24,25]. Van der Ploeg *et al.* [26] observed that low doses of the same Ag NM300K (15 mg Ag/kg soil DW) caused higher effects (for the same mass concentration) than AgNO_3_ in *Lumbricus rubellus* longer term reproduction study. Moreover, also focussing on longer term exposures, (Bicho *et al*., 2015 in preparation) showed that in an *Enchytraeus crypticus* full life-cycle test 20 mg Ag/kg soil DW of Ag NM300K caused an effect equivalent to the reproduction EC50, although the dose response model estimated an EC_50_ = 80 mg Ag/kg soil DW. 

### 4.2. Cellular Level 

#### 4.2.1. AgNO_3_ Mechanisms

Overall, an induction of all measured antioxidant enzymes was observed, with the inhibition of CAT being the exception. Similarly, it has been shown that in *C. riparius*, exposure to AgNO_3_ decreases the CAT activity [27]. Also CuCl_2_ and CuNP have been shown to reduce CAT activity [12], possibly due to direct interaction of Cu with the protein’s thiol groups, altering the tertiary structure of the catalase and inhibiting it [28], possibly with a similar mechanism for Ag. On the other hand, CAT has also been reported activated (in other invertebrates) in the presence of AgNO_3_, e.g., in *Eisenia fetida* [1,4], and in *F. candida* when exposed to copper and cadmium [11]. 

The glutathione-related enzymes, GR and GST present different patterns for activation, GR early and GST later induction. It is known that Ag has a great affinity for thiol groups, besides inducing the production of ROS [29,30,31]. Therefore, the presence of Ag can mobilize the GSH levels in the cell (*i.e.*, binding to this substrate) [32,33], so here it seems that an early activation of GR occurred to compensate the unavailable GSH, *i.e.*, oxidized glutathione. The Ag-GSH detoxification is associated with the GST activation, similar to e.g., the detoxification mechanism of Cd [15], explaining its increase only after 4 days and again after 10 days. Additionally, the initial GR increase followed by a decrease is similar to the response to Cu by *F. candida* [11]. The GST activity and TG content increase after 4 days may be due to ROS generation, this also related with the LPO levels.

The increase in MT levels must be associated with the Ag chelation. This is in agreement with observations at the gene expression level in *E. fetida* exposed to AgNO_3_ [4] and Cu [34], and *F. candida* exposed to Cd [15]. It is known that Ag can be taken up by Cu transporters and interact with Cu homeostasis, which may contribute to Ag toxic effect [29].

Regarding LPO at day 4, this was similar to the response to Cd in *F. candida* [11] and Ag in aquatic invertebrates [35]. This could be the result of the imbalance in the redox in the organisms due to CAT reduced activity, as similarly observed to Cu in *E. albidus* [12]. Such reduced CAT activity leads to accumulation of hydroperoxides, which can be removed via the glutathione cycle enzymes. This is reflected in the initial activation of GR, followed by the increase in GST and TG. When the enzymes activity reach a point of saturation LPO occurs. 

#### 4.2.2. AgNP Mechanisms

In contrast to the AgNO_3_ exposure, CAT activity was significantly increased in the AgNP exposure, except after 6 days, a pattern similarly observed for Cu and Cd in *F. candida* [11]. The MT induction occurred after 6 and 10 days, *i.e.*, later when compared to AgNO_3_. It is unknown if for longer exposure periods this would also be followed by a decrease like in AgNO_3_. 

The increase in the glutathiones (higher GST throughout the exposure length, increased GR after 4 days and the increase in TG after 6-10 days), indicate interactions of AgNP with cytosolic and transmembrane proteins, changing the conformation and impairing the antioxidant defenses [36,37,38,39]. Hence, GST levels were continuously high to chelate the radical ligands in thiol groups in glutathione content [4,30,32,33]. The increase in GR was needed to balance the redox potential (GSH recycling), as a result of ROS production from NP interactions [31]. Because NPs can also cause DNA damage, leading to synthesis of nuclear GSH, this may explain the increase in the TG content [33,40,41].

#### 4.2.3. Comparison of Ag Nano and Ag Salt Mechanisms 

As discussed so far it is clear that Ag nano and Ag salt cause dissimilar oxidative stress mechanisms of response (see Figure 3). Differences in response patterns for AgNO_3_ and AgNP have also been described for e.g., the soil invertebrates *Eisenia fetida* [1,4] and *Enchytraeus albidus* [5]. 

The patterns observed in *F. candida* for GR, TG and MT seem to indicate a delayed effect of AgNP compared to AgNO_3_ (as shown by some authors [42,43]), suggesting an effect caused by the slower release of Ag or a slower uptake On the other hand, CAT and GST show clearly different patterns, indicating a specific NP effect. As already suggested, AgNPs uptake may be done by different pathways than AgNO_3_ [29,31,44,45]. There seems to be a combined effect of Ag^+^ and AgNPs which results in a different time of occurrence of events and consequently a different cascade. This is corroborated by the differences caused in terms of LPO, reflecting previous variations in REDOX enzymes. For instance, following the hypothesis of the Ag^+^ release from AgNPs the response of MT, GR and TG could be seen as a delayed response for the AgNP, however this is not the case for CAT, LPO and GST. 

## 5. Conclusions

Oxidative stress was studied for the first time in *F. candida* to AgNPs. Reproduction effect concentrations (EC50) caused dissimilar oxidative stress mechanisms, indicating a combined effect of released Ag^+^ (MT and GST) and of AgNPs specifically (CAT, GR, TG, LPO). Ag NM300K were less toxic than AgNO_3_ in terms of population effects, *i.e.*, survival and reproduction. 
